# Communication, orientation, coordination: A model for a laparoscopy team

**DOI:** 10.4274/jtgga.2017.0138

**Published:** 2018-03-01

**Authors:** Daniel A. Tsin

**Affiliations:** 1 Former Director of Minimally Invasive Surgery. The Mount Sinai Hospital of Queens, Long Island City, New York, USA

To the Editor;

There are many residency programs, courses, congresses, and articles on training laparoscopists, but very few on developing teams. Communication, Orientation, Coordination (COC) is simple team training for the gradual and safe advancement in laparoscopy and natural orifice transluminal endoscopic surgery. From 1991 until 2003 at today’s Mount Sinai of Queens, New York, we began special sessions for training teams of general surgeons, gynecologists, urologists, and assistants for video laser laparoscopy. We trained on simulators and in the animal laboratory. For lasers, we practiced in accordance with what later would become the United States of America (USA) Occupational Safety and Health Administration guidelines. The communication and coordination that we learned from laser safety complemented the COC. For video laparoscopy, we used our years of experience in laparoscopic gynecology to teach less experienced surgeons and assistants to follow the commands of the surgeon. Some beginners had a tendency to direct the laparoscope opposite to the surgeon’s request and had difficulties with depth perception. We added concepts from football, also known as soccer, and the aviation cockpit or Crew Resource Management (CRM). 

Football is the most popular sport in the world and most fans are familiar with its signs and verbal commands. Coaches establish communication among players as part of the collective strategy and tactics for the team. Players learn from repetitive actions, and short verbal communications are used when possible during a game. However, in laparoscopy there is time for verbal commands.

Since 1979, CRM has continued to evolve, a dedicated paper on this subject is available for free on ResearchGate (1). We focused on the communication in the cockpit between the captain and the first and second officer, which is similar to surgeons and assistants, using the analogy that the surgeon is the captain. Cockpit Voice Recorders (CVR) are reviewed by dedicated committees after unusual events or accidents; we used a video recorder instead of the CVR. It is important to review our recorded surgeries and compare them with expert videos. In 1997, we began training teams for more advanced procedures. The orientation problems with depth perception were overcome by experience. Next, we had to overcome large off-axis views. Orientation was more difficult when the angle increased from 0° to 180° in relation to the working area. Orientation was achieved by changing forms of communication in these situations. The commands are given as topographic directions rather than directional movements. Additionally, we limited the higher angles off-axis to a specific step or shorter parts of the operation. Disorientation is tiresome, it delays procedures and increases risk. COC team training promotes sharing knowledge, experience, expense, and equipment. Like in football, we need good players and a good team to excel. In my opinion, COC training was time well spent.
